# Cost-Effectiveness of Saxagliptin Compared With Glibenclamide as a Second-Line Therapy Added to Metformin for Type 2 Diabetes Mellitus in Ethiopia

**DOI:** 10.1177/23814683211005771

**Published:** 2021-04-27

**Authors:** Mengistu Bekele, Ole Frithjof Norheim, Alemayehu Hailu

**Affiliations:** Bergen Center for Ethics and Priority Setting, Department of Global Public Health and Primary Care, University of Bergen, Bergen, Norway; Oromia Regional Health Bureau, Addis Ababa, Ethiopia; Bergen Center for Ethics and Priority Setting, Department of Global Public Health and Primary Care, University of Bergen, Bergen, Norway; Department of Global Health and Population, Harvard T.H. Chan School of Public Health, Boston, Massachusetts; Bergen Center for Ethics and Priority Setting, Department of Global Public Health and Primary Care, University of Bergen, Bergen, Norway

**Keywords:** glibenclamide, metformin, saxagliptin, second-line diabetes mellitus treatment, type 2 diabetes mellitus

## Abstract

**Background.** Metformin is a widely accepted first-line pharmacotherapy for patients with type 2 diabetes mellitus (T2DM). Treatment of T2DM with glibenclamide, saxagliptin, or one of the other second-line treatment agents is recommended when the first-line treatment (metformin) cannot control the disease. However, there is little evidence on the additional cost and cost-effectiveness of adding second-line drugs. Therefore, this study aimed to estimate the cost-effectiveness of saxagliptin and glibenclamide as second-line therapies added to metformin compared with metformin only in T2DM in Ethiopia. **Methods.** This cost-effectiveness study was conducted in Ethiopia using a mix of primary data on cost and best available data from the literature on the effectiveness. We measured the interventions’ cost from the providers’ perspective in 2019 US dollars. We developed a Markov model for T2DM disease progression with five health states using TreeAge Pro 2020 software. Disability-adjusted life year (DALY) was the health outcome used in this study, and we calculated the incremental cost-effectiveness ratio (ICER) per DALY averted. Furthermore, one-way and probabilistic sensitivity analysis were performed. **Results.** The annual unit cost per patient was US$70 for metformin, US$75 for metformin + glibenclamide, and US$309 for metformin + saxagliptin. The ICER for saxagliptin + metformin was US$2259 per DALY averted. The ICER results were sensitive to various changes in cost, effectiveness, and transition probabilities. The ICER was driven primarily by the higher cost of saxagliptin relative to glibenclamide. **Conclusion.** Our study revealed that saxagliptin is not a cost-effective second-line therapy in patients with T2DM inadequately controlled by metformin monotherapy based on a gross domestic product per capita per DALY averted willingness-to-pay threshold in Ethiopia (US$953).

## Introduction

The burden of type 2 diabetes mellitus (T2DM) has steadily increased over the past couple of decades across the globe. In 2017, nearly half a billion people were affected by diabetes, and about 70% of those patients were living in developing countries.^1^ Ethiopia is one of the countries that are profoundly affected by T2DM, where it is one of the leading causes of death and complications.^2,3^ For example, a study from the northern part of the country shows that the majority of T2DM patients presented to a clinic with some sort of complication.^4,5^ Another hospital-based study revealed that 70% of T2DM patients had experienced at least one of the chronic complications.^6^

T2DM places a substantial economic burden on households and health systems in low-income countries.^7,8^ This burden can be related either to direct costs incurred by society in managing the disease and its complications or to indirect costs resulting from productivity losses due to disability and premature mortality as well as time spent by family members when accompanying patients seeking care. For instance, according to a recent systematic review, on average, T2DM costs US$7 per each outpatient visit, and each patient incurs an additional cost of US$290 for inpatient stays, US$25 for laboratory tests, and US$177 for medications every year.^8^

The control of blood glucose is a crucial intervention in the management of T2DM patients, and there are effective treatment modalities. At the initial stage, dietary modification and physical activity are recommended for adequate glycemic control. Metformin is effective and is the most widely used first-line pharmaceutical therapy for T2DM that is uncontrolled by lifestyle management. Glibenclamide has long been the mainstay of diabetic management when the first-line treatment is unable to control the glucose level and reduce the incidence of complications. Finally, insulin is recommended as a third-line treatment.^9,10^

Saxagliptin is one of the recently introduced second-line drugs with substantial additional health benefits in terms of improved glycemic control and low incidence of diabetes-related complications.^9,11,12^ Some cost-effectiveness studies are comparing saxagliptin to glibenclamide as second-line drugs, but almost all the studies are from high-income settings,^13–19^ and most of them were not conducted by an independent researcher.^9^ According to those studies, using saxagliptin as a second-line drug is a cost-effective alternative.

In Ethiopia, some private health facilities and a few public facilities introduced saxagliptin as an alternative drug for their patients without adequate evidence of its additional benefits compared with the additional cost of the drug. The need for transparent, evidence-based country-level data on diabetes management protocols is great in order to allocate scarce health resources efficiently.^20,21^ However, no study has yet compared the cost-effectiveness of the second-line drugs in Ethiopia, and no published study exists from another low-income setting. Therefore, this study aimed to estimate the cost-effectiveness of saxagliptin and glibenclamide as second-line therapies added to metformin compared with metformin only in T2DM in Ethiopia.

## Methodology

### Study Design and Setting

This cost-effectiveness study was conducted in Ethiopia in 2019. We used a mix of primary data from Tikur Anbessa Specialized Hospital (TASH) on cost and the best available data from the literature on the effectiveness of the treatment modalities. TASH is affiliated with the Addis Ababa University College of Health Sciences, and it is the largest specialized hospital in Ethiopia, with a 560-bed capacity.^22^ It provides health care services for about 370,000 to 400,000 people annually. In 2015–2016, about 2800 T2DM patients visited the endocrinology unit of TASH. Endocrinology is a specialty service rendered under the internal medicine department.^23^ Diabetic patients had more inpatients days, with an average length of stay of 10 days, than the 5.7 days for patients with other types of disease.^24^

### Description of the Compared Interventions

In this study, we compared two second-line drugs against each other and against the base-case scenario of routine intervention, which is to provide metformin alone, even in uncontrolled T2DM. Therefore, the first intervention was to add glibenclamide to metformin (metformin + glibenclamide) when a patient’s blood glucose level was inadequately controlled by the maximum dose of metformin alone. In general, adding drugs belonging to the sulfonylurea group (i.e., glibenclamide) is very common.^25^ The second intervention was adding saxagliptin to metformin (metformin + saxagliptin) when a patient’s blood glucose level was inadequately controlled by the maximum dose of metformin alone. Saxagliptin is a new oral hypoglycemic agent, and it has demonstrated significant glycemic-lowering effects. It is weight neutral, well tolerated, and has a low risk of hypoglycemia.^26^ It is also less expensive than other dipeptidyl peptidase-4 inhibitors.^27^ The third intervention was drug treatment with metformin only (the routine intervention). We assumed maximal doses of metformin (2 g/day).

Neutral protamine Hagedorn insulin was added after metformin + glibenclamide or metformin + saxagliptin treatments failed as second-line treatment alternatives. Rather than adding insulin to the oral drugs, we withdrew the second-line oral drugs, and intensive insulin therapy was started as the insulin provided a similar effect exogenously. The drug cost was calculated based on the defined daily dose from World Health Organization (WHO) recommendations.^9,10^ In Table 1, we summarize the interventions.

**Table 1:** Description of the Interventions

Treatment Strategy	Description of the Intervention
Metformin + saxagliptin	Metformin 500 mg four times daily and saxagliptin 5 mg once daily
Metformin + glibenclamide	Metformin 500 mg four times daily and glibenclamide 5 mg twice daily
Metformin only	Metformin 500 mg four times daily

### Cost-Effectiveness Modeling

**Figure 1:**
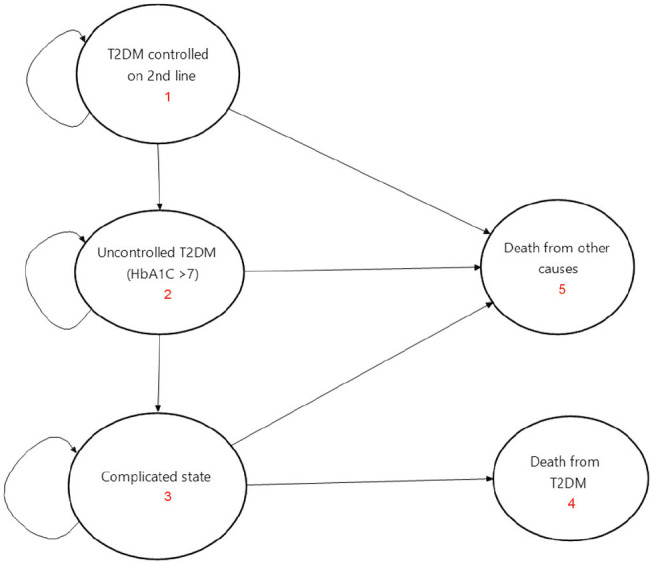
Markov state transition diagram.

Using TreeAge Pro 2020, we developed a Markov process model for T2DM disease progression to calculate the clinical outcomes and costs during the life cycles of people under alternative treatment scenarios (Figure 1). We populated the model with appropriate effectiveness and cost data, either from primary analysis or from the best available sources. Five mutually exclusive health states were used in the model. Each of the states represents the dynamics of T2DM.

The health states are the following: 1) T2DM controlled with second-line treatment (HbA1C <7), 2) uncontrolled T2DM (HbA1C >7.0), 3) complicated T2DM, 4) death from T2DM, and 5) death from all other causes. According to this model, initially, all individuals were in the “T2DM controlled with second-line treatment” state. Then, a person from this initial state could progress to “uncontrolled T2DM” when the HbA1C rose above 7.0 with a certain probability. At this stage, a patient might develop one of the complications with a certain probability and eventually progress to “death from T2DM.”

Each state was associated with annual state rewards related to spending a year in a particular health state. These included the annual cost of treatment and the annual effectiveness value in terms of disability-adjusted life years (DALYs) averted. To account for the recurrent nature of complications due to T2DM, we accounted for complications due to T2DM both as an event and as a state.^28^

The majority of people affected by T2DM are at the age of 40 years and above. Therefore, in this model, we followed a hypothetical Ethiopian population cohort from age 40 over their lifetimes (i.e., an adult population with T2DM controlled with one of the second-line treatments). Thus, the time horizon in this evaluation was 40 years. A similar Markov lifecycle cohort model was employed for each intervention group.

**Figure 2:**
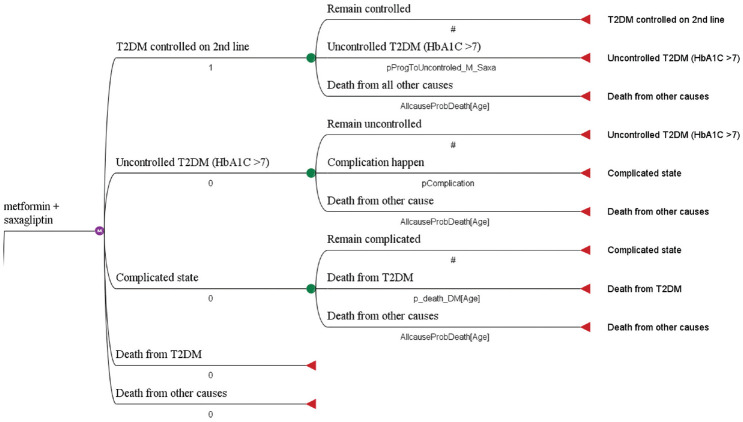
Markov tree diagram for the model.

Transition probabilities were used to capture the probabilities of moving from one state to another (within a specific period called cycle length). Taking into account that T2DM is a chronic disease, a 1-year cycle length was applied in this model. A half-cycle correction was done to assume that events occur halfway through a cycle (rather than at the beginning or the end; Figure 2).

### Measurement of Interventions Cost

The identification, measurement, and valuation of the cost of the intervention and the cost of T2DM diagnosis and treatment were conducted from the providers’ perspective. Household out-of-pocket expenditures, the cost of premature mortality from T2DM, productivity loss for T2DM, and the cost of lost productivity by accompaniers or caregivers to the T2DM patients were not accounted for in this study.^29^

All the cost inputs needed to treat patients whose glycemic level was inadequately controlled (documented through a biochemical test with hemoglobin A1C greater than 7.5), including the addition of other oral antidiabetic regimens to metformin, were collected from TASH. The direct medical costs included clinical staff time allocated to each intervention, pharmaceutical and nonpharmaceutical supplies, and diagnostic tests, oral hypoglycemic medicines, and insulin. The overall cost was limited to the total cost of metformin, glibenclamide, saxagliptin, insulin, outpatient consultation, laboratory consumables, and complications management. Four outpatient clinic visits, four comprehensive laboratory tests per patient, and the administration of the maximum tolerated doses of each drug were assumed.

The cost data were collected prospectively. All the direct medical costs for uncomplicated T2DM management were accounted for. We used a spreadsheet to record the cost information needed for patient treatment, accessing all the available records in the hospital from the second week of February 2019 to the end of the month. The type and quantity of each resource used in the intervention were registered. We captured the economic costs of the interventions (whether they incurred a financial expenditure or not). For example, the time spent by the health personnel involved in treating the patient was accounted for.

To identify the economic value of the resources used, we used the lowest purchasing price for most of the materials and equipment, including the drugs and supplies. For items for which the price was not known from the invoice or the available records, we used estimated values for the items from market inventory data. The unit price of saxagliptin was taken from a wholesale private drug importing company in Addis Ababa.

The valuation of personnel cost was based on an estimated proportion of working time spent on treating a diabetic patient. The personnel cost included the cost of health professionals’ time who were involved in treating a patient with T2DM. The cost of the consultation was collected by normative costing, by consulting standard treatment guidelines, and from professionals involved in case management. Normative costing is a bottom-up costing method that involves estimating resource use for various services by using guidelines and norms when the services are not available. It is recommended when detailed service cost information is not available or is thought to be highly distorted. It is usually used for T2DM case management.

All the costs for the services rendered to T2DM patients whose glycemic level was inadequately controlled by metformin accrued in the respective treatment strategies of the model to calculate an average cost of the treatment of one patient for a year (Table 2).

**Table 2:** Summary of Costs and Costing Method for Second-Line Type 2 Diabetes Mellitus Treatment

Type of cost	Identification	Measurement	Valuation
Personnel	Nurse, pharmacist, phlebotomist, laboratory technologist, general practitioner, internist/endocrinologist, ophthalmologist, surgeon	Number of full working days spent on patient care	Salary information
Drug and supplies	Metformin, glibenclamide, saxagliptin, insulin, insulin syringe, reagents for organ function test, glucometer, chemistry machine	The quantity consumed (in appropriate units) from receipts, pay bills, logbooks	Purchasing price of the item directly from the invoice or the current market price of the item when the invoice was not available

The cost of an intervention was calculated for 40 years with a discount rate of 3% per annum. It is expressed in US dollars, and the average 2019 Ethiopian birr-to-dollar exchange rate has been applied. The drug cost per unit item is as follows: metformin US$ 0.019/tablet, glibenclamide US$ 0.006/tablet, saxagliptin US$ 0.65/tablet, and neutral protamine Hagedorn insulin US$ 0.006/IU. Daily treatment costs were weighted using the daily dose to reflect an average annual consumption. All the costs were calculated in US dollars (Supplement File 1). Microsoft Excel was used to analyses all the cost data.

### Measurement of Health Effects

DALYs averted were used as the health outcome measure (effectiveness). The DALY estimate combines the years of life lost (YLLs) due to premature death and the years of life lived with disability (YLDs).^30^ The YLDs were calculated using a health utility for uncomplicated T2DM of 0.78 and a health utility for complicated T2DM of 0.726. Taking into account the disability weights for diverse types of diabetic complications, we assumed an average additional utility decrement of 0.13 if an event of complications happened.^13,31,32^

Deaths due to controlled T2DM are rare, so we assumed zero mortality. Uncontrolled T2DM might progress to the complicated state, and death from complicated T2DM states may happen. However, because death from T2DM varies across ages, we took different probabilities for the different age groups (Table 3).

**Table 3:** Probabilities and Cost Inputs Used in the Cost-Effectiveness Analysis Model (2019 US Dollars)

Parameters	Mean	Min.	Max.	SD	Dist.	Source
Cost of treatment: metformin + glibenclamide	75	60	90	7.5	Gamma	Primary
Cost of treatment: metformin + saxagliptin	309	247.2	370.8	30.9	Gamma	Primary
Cost of treatment: metformin only	70	56	84	7.0	Gamma	Primary
Average cost of complications (per event)	228	182.4	273.6	22.8	Gamma	44^a^
Cost of metformin + insulin	208	166.4	249.6	20.8	Gamma	Primary
Probability of progression from uncomplicated to complicated while on metformin + glibenclamide	0.053	0.04	0.06	0.01	Beta	38^a^
Probability of progression from uncomplicated to complicated while on metformin + saxagliptin	0.013	0.01	0.03	0.00	Beta	38^a^
Probability of progression from uncomplicated to complicated while on metformin only	0.122	0.10	0.15	0.01	Beta	39^a^
Probability of complication while on metformin + insulin	0.4	0.32	0.48	0.04	Beta	Ass.
Disutility: complicated T2DM	0.274	0.22	0.33	0.03	Beta	13, 31, 32^a^
Disutility: uncomplicated T2DM	0.220	N/A	N/A	N/A	Beta	13, 31^a^
Disutility from death	1	N/A	N/A	N/A	N/A	GBD
Utility decrement because of one complication event	0.13	N/A	N/A	N/A	N/A	31
Initial age (years)	40	N/A	N/A	N/A	N/A	Ass.
Discount rate health utility (%)	3	0	5	N/A	N/A	33
Discount rate cost (%)	3	0	5	N/A	N/A	33
Number of cycles	40	N/A	N/A	N/A	N/A	45

Ass., assumption based on expert opinion; Dist., distribution; GBD, Global Burden of Disease study; Max., maximum value; Min., minimum value; N/A, not applicable; SD, standard deviation.

a Studies used as secondary source of input data for this model were appraised using appropriate checklist.

### Cost-Effectiveness Analysis

The incremental cost-effectiveness ratio (ICER), cost-effectiveness scatterplot, and cost-effectiveness acceptability curve were produced using TreeAge Pro 2020 software to summarize and present the cost-effectiveness results.^29^ The expected costs and health outcomes (DALYs averted) were calculated for each of the three treatment options. We ranked all the interventions in ascending order in terms of cost of intervention, and each intervention was thus compared with the next costliest intervention to calculate the incremental costs, the incremental effectiveness, and the ICER. We eliminated from comparison the interventions that cost more but provided fewer benefits than an alternative intervention (dominance).

#### Probabilistic Sensitivity Analysis

Overall model uncertainty was analyzed with probabilistic sensitivity analyses (PSA) using a Monte Carlo simulation, and the results are presented as cost-effectiveness acceptability curves, cost-effectiveness acceptability frontiers, and scatterplots. A Markov chain Monte Carlo simulation with 100,000 iterations was done using TreeAge Pro 2020 software. In the PSA, the variables in the model were replaced with distributions. Probabilistic distributions for costs, disutilities, and transition probabilities were assigned with most likely (mean), minimum (min.), and maximum (max.) values. We assumed those cost parameters to have a gamma distribution and the health outcome and transition probabilities to follow a beta distribution. We considered the minimum and maximum transition probabilities to vary ±5% from the most likely values. We considered the minimum and maximum intervention costs to vary ±20% from the most likely values (Table 3).

#### One-Way Sensitivity Analysis

To test the robustness of the model’s conclusion to some of the assumptions, we performed one-way sensitivity analyses on all the costs (i.e., cost of treatment with metformin + saxagliptin, cost of treatment with metformin + glibenclamide, cost of treatment with metformin only, cost of insulin, and cost due to complications) and transition probabilities (Table 3). We did this for different levels of cost and effectiveness parameters, and we present the results in a tornado diagram. Additionally, variables such as time horizon, cost, the probability of complications from T2DM, the probability of progression to uncontrolled T2DM while on metformin + glibenclamide, the probability of progression to uncontrolled T2DM while on metformin + saxagliptin, the health utility of uncomplicated T2DM, the health utility of complicated T2DM, the probability of having uncontrolled T2DM while on metformin only, the utility decrement because of disability, and the probability of complications while on metformin + insulin were included in the one-way sensitivity analysis.

#### Cost-Effectiveness Thresholds

Based on the economic theory of maximization of the expected health benefits from the interventions, the optimal decision is to choose the strategy with the highest ICER per DALY averted that falls just at or below the willingness-to-pay (WTP) threshold.^33^ In light of that, there are different recommendations for cost-effectiveness thresholds (CETs). The WHO’s Choosing Cost-Effective Interventions (CHOICE) describes interventions with an ICER per DALY averted of less than one times the gross domestic product (GDP) per capita of the country as “very cost-effective,” of one to three times the GDP per capita as “cost-effective,” and of greater than three times the GDP per capita as “not cost-effective.”^34^ However, recent empirical evidence indicates that the GDP per capita is still high as a CET, and therefore a new recommendation is to use 50% of the GDP of a country as a reference. Therefore, in this study, we applied a CET threshold of 50% of GDP per capita. Based on Ethiopia’s GDP per capita for the year 2019 of US$953, a CET of US$476.5 was used in this study.^35^

## Results

The annual unit cost of metformin was US$70. The annual unit cost of metformin + glibenclamide was US$75. The annual unit cost of metformin + saxagliptin was US$309. Similarly, the annual unit cost of metformin + insulin was US$208. The average unit cost per event of one of the complications was US$228. The annual unit cost of insulin was US$208 (Table 4).

**Table 4:** Unit Costs of the Interventions (2019 US Dollars)

Cost	Mean
Intervention cost of metformin	70
Intervention cost of metformin + glibenclamide	75
Intervention cost of metformin + saxagliptin	309
Average cost of complications (event)	228
Cost of insulin	208

The expected costs per person for the three T2DM treatment options were US$3603.30, US$1733.10, and US$1449.70 for metformin + saxagliptin, metformin only, and metformin + glibenclamide, respectively. Metformin + saxagliptin was almost two times more costly than metformin + glibenclamide. However, in terms of health effects, the expected DALY among those who were on metformin + saxagliptin was only 14.413 DALYs. The expected DALY among those on metformin only was 16.296, and that among those who were on metformin + glibenclamide was 15.366. Metformin only was more costly and less effective than metformin + glibenclamide. Metformin only was strongly dominated by metformin + glibenclamide and, therefore, excluded from further comparison in the model. In general, the model predicts that the ICER for metformin + saxagliptin will be US$2259 per DALY averted compared to metformin + glibenclamide (Table 5).

**Table 5:** Cost, Effectiveness, and ICER in 2019 US Dollars

Strategy	Cost	Incremental Cost	Effectiveness (DALYs)	Incremental Effectiveness	ICER	ACER
Excluding dominated						
Metformin + glibenclamide	1449.7		15.366			94
Metformin + saxagliptin	3603.3	2153.6	14.413	0.953	2259	250
All						
Metformin + glibenclamide	1449.7	0.0	15.366	0.000		94
Metformin only (routine)	1733.1	283.4	16.296	−0.929	−304	106
Metformin + saxagliptin	3603.3	2153.6	14.413	0.953	2259	250

ACER, average cost-effectiveness ratio; DALY, disability-adjusted life year; ICER, incremental cost-effectiveness ratio.

a Effectiveness is measured in DALYs (fewer DALYs are better than more DALYs).

**Figure 3:**
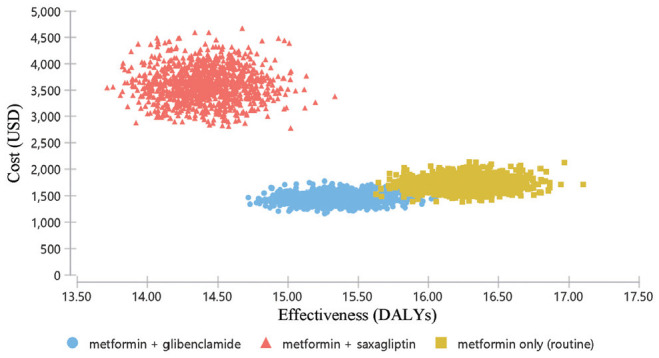
Cost-effectiveness scatter plot.

The cost-effectiveness scatterplot indicates that there was wide variability across both the cost and effectiveness of metformin + saxagliptin, while the variability in the other two options was mainly across the effectiveness dimension (Figure 3).

**Figure 4:**
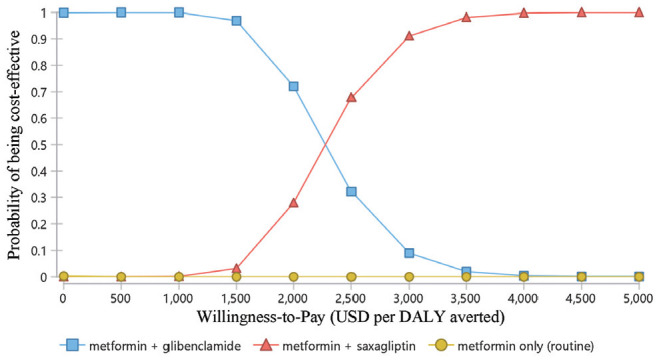
Cost-effectiveness acceptability curves.

The cost-effectiveness acceptability curves (Figure 4) indicate the probability of being cost-effective at various levels of WTP per DALY averted. For example, the probability of being a cost-effective option of metformin + saxagliptin was less than 5% at a WTP threshold of US$953 per DALY averted (one times GDP per capita), while at a WTP threshold of US$2859 per DALY averted (three times GDP per capita), the probability of metformin + saxagliptin being a cost-effective option was slightly above 80%.

**Figure 5:**
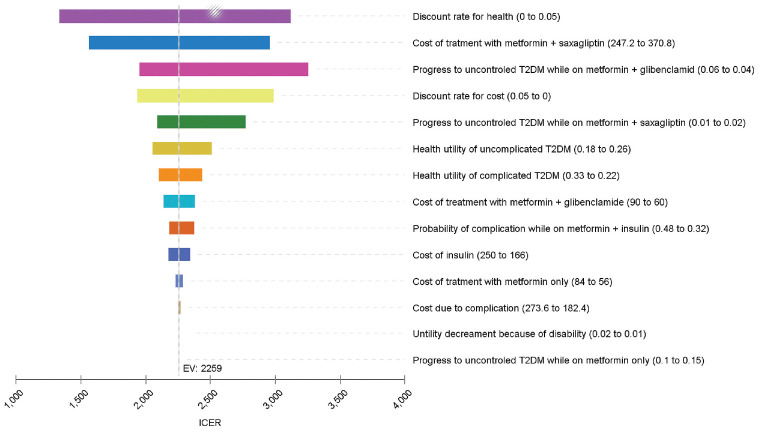
Tornado diagram.

One-way sensitivity analysis was conducted by varying the range of values to determine the potential impacts on the results. The cost of treatment with metformin + saxagliptin, progression to uncontrolled T2DM while on metformin + glibenclamide, probability of uncontrolled T2DM while on metformin + saxagliptin, health utility of uncomplicated T2DM, health utility of complicated T2DM, and cost of treatment with metformin + glibenclamide in the model had the most substantial impact on the ICER results. However, the ICER result was less sensitive to changes in the probability of having uncontrolled T2DM while on metformin only, in utility decrement because of disability, in cost due to complication, in cost of treatment with metformin only, in probability of complication while on metformin + insulin, and in the cost of insulin (Figure 5).

**Figure 6:**
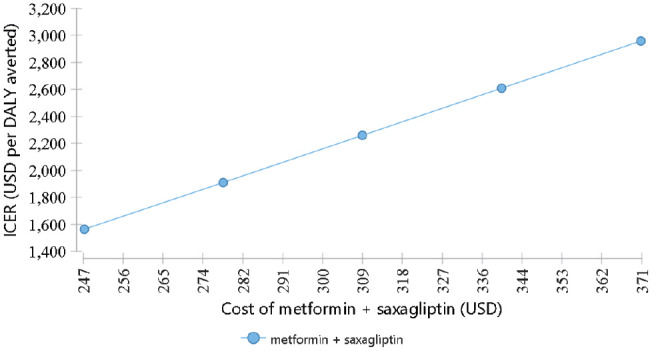
One-way sensitivity analysis of the cost of metformin + saxagliptin.

In a one-way sensitivity analysis, we tested the effect of the cost of metformin + saxagliptin on the cost-effectiveness of the metformin + saxagliptin option by varying the annual cost from US$245 to US$380 while keeping all other variables at their base-case values. The results show that the ICER for the saxagliptin option remains above the CET even if the cost of metformin + saxagliptin is reduced by 20% from the mean value (US$309; Figure 6).

**Figure 7:**
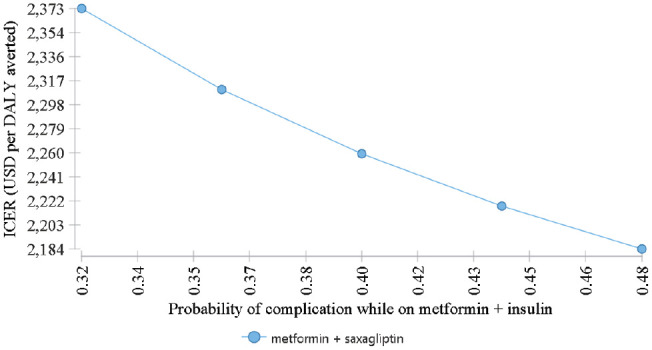
One-way sensitivity analysis for the probability of complication while on metformin + insulin.

We present the one-way sensitivity analysis in Figure 7. When the probability of complication while on metformin + insulin vary from the mean value (40%) to lowest (32%) and the highest (48%), the ICER for metformin + saxagliptin would vary only from the lowest US$2180 to the highest about US$2380 per DALY averted.

## Discussion

This study estimates the cost-effectiveness of saxagliptin as a second-line therapy in Ethiopia. We found a baseline ICER of US$2259 for metformin + saxagliptin. We also found that metformin monotherapy is strongly dominated by metformin + glibenclamide (i.e., metformin monotherapy is more costly and less effective compared with metformin + glibenclamide in the long term) and therefore excluded it from the comparison. This implies that, if the Ethiopian Ministry of Health decided to introduce saxagliptin as a second-line treatment into the health service package instead of glibenclamide, an additional cost of US$2259 would be incurred per each DALY averted using this treatment. Therefore, this study indicates that although metformin + saxagliptin averts more DALYs than metformin + glibenclamide therapy, it is not a cost-effective alternative based on a CET of 0.5 times the GDP per capita of Ethiopia in 2019.

The Ethiopian economy’s capacity is minimal, and so is its health system capacity. This drug, therefore, is not within the “cost-effective range” in Ethiopia. However, the ICER for saxagliptin that we found in this study (US$2259) is relatively low compared with the findings of most studies conducted in other countries. For example, a study from Canada by Klarenbach et al. reported an ICER of US$12,757 per QALY.^13^ Kwon et al. reported an ICER of US$19,420 per life-year gained in the United States.^14^ Another study, from Sweden, by Granström et al., reported an ICER of US$19,348 per QALY.^15^ A study from Argentina concludes that saxagliptin is a cost-effective option with an ICER per QALY of US$7374.^18^ A study from Germany also reports that saxagliptin is cost-effective with an ICER of €13,931 per QALY gained.^16^ A study from China also positively concludes that using saxagliptin is a cost-effective option with an ICER of ¥43,883 per QALY (equivalent to about US$6000).^19^

Our one-way sensitivity analysis also indicates that the cost of saxagliptin is the most crucial variable that influences the ICER for saxagliptin + metformin intervention. The relatively lower cost of saxagliptin in our study may be because of two main reasons. First, the price of reagents, supplies, and drugs (saxagliptin included) is less costly in Ethiopia because the majority of drugs and supplies are usually locally manufactured or imported from the Indian market, which is relatively cheap compared with other markets. For instance, a survey conducted in 2013 to measure and compare the price and availability of locally produced and imported medicines revealed that, of all drugs and supplies found in the outlets, about 55% were locally manufactured products and about 18% were imported from India. About 7% were imported from Cyprus.^36^ Second, the cost of personnel is also relatively low in Ethiopia because the salary paid to health care workers in Ethiopia is low, even compared with other sub-Saharan African countries. Therefore, the personal (human resources) unit cost is low in Ethiopia.^37^

### Limitations

This economic evaluation of saxagliptin as a second-line treatment for T2DM is one of the few from low-income countries and the first from Ethiopia. In this model, to competently represent the country context, the cost inputs were taken either from the primary source or from other good-quality studies conducted in a low-income setting with a context similar to that of Ethiopia. However, the cost information applied in this model has some limitations. For instance, in this study, we considered direct costs only from a provider’s perspective. We did not include nonmedical costs, productivity lost, and costs to other sectors. Also, the costs included in this analysis were obtained from a single hospital-based study at TASH. Although TASH is one of the larger hospitals in the country, serving people from all over Ethiopia, it may not be representative. If cost information from other hospitals in the country had been included, the overall unit cost estimates would most likely have been even higher than the cost of saxagliptin in this study and, therefore, the ICER for saxagliptin + metformin would most likely be slightly higher than US$2259 per DALY averted. We therefore recommend that the Federal Ministry of Health of Ethiopia should establish a nationally representative cost and price database for drugs and medical/health service that can be used for studies of such kind or other key strategic and operational decisions (e.g., reimbursement, budgeting, planning, price negotiation).

To estimate the effectiveness of the drugs, we derived most of the inputs (i.e., utilities and probabilities) for the model from studies conducted outside of Ethiopia. For instance, the disease progression probabilities were from extensive studies conducted on a US population.^38,39^ The health utility inputs were from the UK Prospective Diabetes Study (UKPDS) studies. Although the UKPDS is one of the more robust and widely applied diabetes models for cost-effectiveness evaluation, its application to the Ethiopian context may be uncertain because of variations in the diabetic population between the United Kingdom and Ethiopia.^40^ Therefore, the treatment effects might not be fully transferable to the Ethiopian context because of difference in age structure, other comorbidity, life expectancy, and so on, between the two populations.^41,42^ In principle, this would affect the generalizability of the cost-effectiveness findings.^41,42^ However, the probabilities and one-way sensitivity analysis findings show that the overall effect of change in those parameters on the overall ICER was minimal (Figures 4 and 5).

Furthermore, because of the lack of input data, the Markov process model we applied in this study does not depict the progression of T2DM in detail. For instance, we were not able to represent various types of diabetic complications separately.^43^ Instead, we merged all the complications as one state or one type of event. This limitation is, to some extent, a misrepresentation of the natural progression of the disease. Additionally, we were not able to account for intermediate treatment outcomes, such as hypoglycemia incidence, weight gain, patient satisfaction, and so on. One approach to address these kinds of limitations is to employ discreet event simulation, which would enable us to capture more variables without aggregation. The discreet event simulation would improve the precision of our results. However, we tried to mitigate the impact of such limitations on the overall findings using a PSA and one-way sensitivity analysis, so they are less likely to change the direction of our conclusion.

## Conclusion

The ICER for saxagliptin + metformin was US$2259 per DALY averted. Therefore, our study revealed that the addition of saxagliptin to metformin was not a cost-effective second-line therapy in T2DM patients inadequately controlled by metformin monotherapy based on a WTP threshold of 50% of the GDP per capita in Ethiopia. The ICER was driven primarily by the higher cost of saxagliptin relative to glibenclamide.

## Supplemental Material

sj-docx-1-mpp-10.1177_23814683211005771 – Supplemental material for Cost-Effectiveness of Saxagliptin Compared With Glibenclamide as a Second-Line Therapy Added to Metformin for Type 2 Diabetes Mellitus in Ethiopia Click here for additional data file. Supplemental material, sj-docx-1-mpp-10.1177_23814683211005771 for Cost-Effectiveness of Saxagliptin Compared With Glibenclamide as a Second-Line Therapy Added to Metformin for Type 2 Diabetes Mellitus in Ethiopia by Mengistu Bekele, Ole Frithjof Norheim and Alemayehu Hailu in MDM Policy & Practice
