# Supplementary material for: Cost-Effectiveness of Saxagliptin Compared With Glibenclamide as a Second-Line Therapy Added to Metformin for Type 2 Diabetes Mellitus in Ethiopia
Source: MDM Policy Pract. 2021 Apr 27;6(1):23814683211005771. doi: 10.1177/23814683211005771 (PMC8111283; doi:10.1177/23814683211005771)
Supplement: sj-docx-1-mpp-10.1177_23814683211005771 – Supplemental material for Cost-Effectiveness of Saxagliptin Compared With Glibenclamide as a Second-Line Therapy Added to Metformin for Type 2 Diabetes Mellitus in Ethiopia [file sj-docx-1-mpp-10.1177_23814683211005771.docx]

## Supplement file 1. Unit prices of drugs, supplies, tests, and aannual personnel cost per health professional (2019 Ethiopian Birr (ETB)) at TASH.

| Items needed | Unit Price (in ETB) |
| --- | --- |
| Unit prices of drugs, supplies, tests | |
| Blood glucose test | 10.00 |
| Urine analysis | 10.00 |
| Glibenclamide, 5 mg tab | 0.14 |
| Metformin, 500 mg tab | 0.42 |
| Saxagliptin, 5 mg (per tab) * | 14.40 |
| NPH insulin (per ml) | 13.60 |
| Insulin syringe | 1.10 |
| Glucose strips | 8.40 |
| Glucose dipstick (per test) | 1.40 |
| Lipid profile | 40.00 |
| Liver function test | 25.00 |
| Renal function test | 10.00 |
| Electrocardiogram test | 60.00 |
| Echocardiography test | 150.00 |
| Annual personnel cost per health professional at TASH in 2019 ETB | |
| General practitioner | 17,655 |
| Endocrinologist | 26,538 |
| Internist | 26,538 |
| Orthopedic surgeon | 26,538 |
| Senior nurse | 9,028 |
| Anesthetist | 9,028 |
| Ophthalmologist | 26,538 |
| Senior consultant | 37,896 |

* The unit cost data for saxagliptin was taken from a private wholesale pharmacy in Addis Ababa as it was not used in the treatment of patients at TASH.
